# Knowledge and compliance with human immunodeficiency virus post exposure management among dentists in two tertiary hospitals in Lagos, Nigeria

**DOI:** 10.1017/ash.2025.10150

**Published:** 2025-10-10

**Authors:** Ifeoluwa Bridget Falokun, Oyinkansola Olulola Sofola, Omolara Gbonjubola Uti

**Affiliations:** 1 Preventive Dentistry Department, Lagos University Teaching Hospital, Idi-Araba, Lagos, Nigeria; 2 Faculty of Dental Sciences, College of Medicine, University of Lagos, Lagos, Nigeria

## Abstract

**Objectives:**

This study aims to assess the knowledge and determine the level of compliance with human immunodeficiency virus (HIV) post exposure management (PEM) among dentists.

**Design:**

A descriptive cross-sectional study was done among 114 dentists.

**Setting:**

Study was done among dentists in two Nigerian tertiary hospitals.

**Participants:**

All dentists available, who consented to the study during the period of data collection were included in the sample. Three cadres of dentists; house officers, residents and consultants, were involved in the study.

**Methods:**

Using self-administered questionnaires, information was sought on knowledge of managing exposures-first aid and prophylaxis as well as compliance when exposed. Data was analyzed using the SPSS statistical software.

**Results:**

Nearly all respondents, 111(97.4%), reported having heard about HIV PEM. Exposure incidents most frequently identified by respondents were needlestick injuries, 111(97.4%). Majority of the dentists recognized practices such as flushing exposed mucous membranes with water, 97(85.1%), and washing skin injuries with soap and water, 75(65.8%), as first aid for exposure incidents, while 20(17.5%) endorsed inappropriate measures like applying bleach to the wound. Seventy-one (62.3%) reported awareness of a HIV PEM protocol in their institution while 39(33.3%) were uncertain, with only 25(21.9%) reporting routine practice of the protocol. Fifty-six (49.1%) of the respondents had experienced an exposure of which only 30(53.6 %) reported and 32(57.1%) requested blood tests for the source patient(s).

**Conclusion:**

The level of compliance with post exposure management is inadequate, therefore there is a need to update the knowledge and device methods of encouraging compliance with HIV PEM protocol among dentists.

## Introduction

Healthcare professionals, especially dentists, face significant risks of occupational exposure to infectious agents, with human immunodeficiency virus (HIV) being a primary concern.^1^ Exposure to potentially infectious bodily fluids can occur through various routes, including needlestick injuries and contact with mucous membranes or non-intact skin.^1,2^ The dental profession is particularly vulnerable due to frequent contact with blood and saliva during procedures, as well as the routine use of sharp instruments. Proper postexposure management is crucial in reducing the risk of infection following such incidents.^3^ Given the potential consequences of HIV transmission, it has become a focal point in dental safety research and protocols.^4^

Occupational exposure to blood-borne pathogens remains a significant concern in dental practice, with studies across various regions highlighting the prevalence of exposure incidents.^5,6,7^ While the risk of infection varies depending on multiple factors, all exposures to contaminated blood require immediate medical attention. Postexposure management protocols typically involve washing of the affected area with soap and water, comprehensive assessment of the incident, prompt reporting, and voluntary testing of the source individual for HIV.^3^ When indicated, postexposure prophylaxis (PEP) for HIV may be initiated.^8^

In low- and middle-income countries (LMICs), healthcare workers, including dentists, face significant HIV exposure risks. Despite the critical role of PEP in preventing HIV transmission, awareness and utilization remain suboptimal in these regions.^4,9^ Nigeria, with an HIV prevalence of 1.4% and approximately 1.9 million people living with HIV, presents a unique challenge.^10^ Oral manifestations are common among HIV-positive individuals, with a Nigerian study reporting 36.8% of patients presenting oral lesions.^11^ This high prevalence increases the likelihood of dentists encountering HIV-positive patients during treatment. Consequently, dentists must be well-versed in PEP protocols to protect both themselves and their patients, especially given the rare but documented cases of patient infection in dental settings.^12^

While specific data on HIV prevalence among Nigerian dentists is lacking, a previous study revealed that 45% of dentists in Nigeria had active hepatitis B virus infection, as indicated by detectable HBsAg.^13^ This finding underscores the urgent need for improved occupational safety measures and strict adherence to postexposure management protocols in resource-limited settings. The present study aims to assess dentists’ knowledge and compliance with post exposure management in two Nigerian tertiary hospitals, one of which has a HIV antibody seroprevalence of 5.77% among patients in the emergency department.^14^ By identifying barriers to guideline adherence, this research intends to contribute to the development of strategies that enhance workplace safety, improve occupational health practices, and potentially inform policy-making and resource allocation in dental practice across Nigeria and similar resource-constrained environments.

## Methodology

### Site and population

An institution based descriptive cross-sectional study was done among dentists in two tertiary health institutions in Lagos, Nigeria: Lagos University Teaching Hospital (LUTH), Idi-araba, Lagos, Nigeria and Lagos State University Teaching Hospital (LASUTH), Ikeja, Lagos, Nigeria. LUTH was established in 1962 with the Faculty of Dental Sciences established in 1966. LASUTH was originally a cottage hospital commissioned in 1955 but was transformed to a tertiary institution in 1999 with the dental center commissioned in 2008. Cadre of dentists included in the target population were the consultants, residents, and house officers.

### Study design

The study was a descriptive cross-sectional study.

### Data collection

Recruitment of participants and data collection were done simultaneously from July 2024 to September 2024. Data was collected using physically self-administered written questionnaires. Physical questionnaires were stored in a secured locker in protective folders for the period of data collection. A total of 13 questions were designed to assess the knowledge of HIV post exposure prophylaxis (PEM). The items specifically assessed source of information about HIV PEM, dentists’ knowledge on what incidents are referred to as exposure incidents; identification of potentially infectious body fluids; knowledge regarding if all exposures will result in HIV infection; steps in HIV PEM including first aid procedure for an exposure and recommended timing for initiation; knowledge of post exposure prophylaxis (PEP) drug regimen in use and recommended duration. Questions on steps involved in post exposure management and post exposure prophylaxis regimen in use were open-ended. The potential responses for source of information were recorded by “Books,” “Journals,” “colleagues,” “Seminars,” and “others.” The response to one question used to assess if all exposures will result in HIV infection was recorded by “Yes,” “No” and “I don’t know.”

A total of 9 questions were designed to assess compliance with HIV post exposure management. The items specifically began with a contingency question to assess if the dentist had ever been pricked with a sharp instrument previously in contact with a patient’s body fluid. This response was accompanied by a “Yes” or “No” response. Participants who responded “Yes” were then instructed to proceed to other questions including frequency of exposure incidents dentist had experienced in the past one year, if incident(s) was reported, reasons for not reporting incident, if blood tests were requested, HIV status of source patient(s), and commencement of HIV PEP. Questions on reporting of incident, requesting blood tests for source patient and commencement of HIV PEP were accompanied by “Yes” and “No” responses. Reasons for not reporting was an open-ended question. All dentists available, who consented to the study during the period of data collection were included in the sample.

### Data management and analysis

Data entry and analysis were done using the SPSS statistical software version 25. Descriptive statistics like frequencies and proportions were used to summarize the data. Tests for association were done using the Pearson’s *χ* ^2^ tests. Differences were considered statistically significant at the level of *P* < 0.05.

### Ethical considerations

Ethical approval for this study was obtained from the Health Research Ethics Committee of the Lagos University teaching Hospital before questionnaires were administered to the respondents.

## Results

A total of 114 respondents were recruited for this study. The median age of respondents is 30.5 years, with an interquartile range of 27.25 to 36.00 years. Gender distribution in this sample indicates a slight majority of female respondents (Table 1). Nearly all respondents (97.4%) reported having heard about postexposure management with the primary sources of this knowledge being books (61.3%), information obtained from colleagues (57.7%), journals (39.6%), and seminars (49.5%).

**Table 1.** Demographic distribution of respondents

Characteristic	House officer	Resident	Consultant	*n* = 114^1^
**Age group (years)** 20–29 30–39 40–50	32 3 0	12 46 11	0 3 7	44 (38.6%) 52 (45.6%) 18 (15.8%)
**Gender** Female Male	23 12	36 33	5 5	64 (56.1%) 50 (43.9%)

n (%).

Exposure incidents most frequently identified by respondents are needlestick injuries, recognized by 97.4%, followed by cuts with sharp objects (77.2%) and splashes to mucous membranes or open skin (73.7%). Thirty-six percent of respondents wrongly categorized contact of intact skin with blood as an exposure. Majority (91.2%) correctly understood that not all exposures will result in infection (Table 2).

**Table 2.** Study participants’ responses to questions about HIV post exposure management

Question	Response	Frequency (*n* = 114^1^)
The following are potentially infectious body fluids^a^	Blood Saliva Gingival fluid All of the above	31(27.2%) 18 (15.8%) 5 (4.4%) 84 (73.7%)
Do all exposures result in infection?	Yes No I don’t know	3 (2.6%) 104 (91.2%) 7 (6.1%)
What first aid measures should be taken following an exposure incident?^a^	Wash injuries to skin with soap and water Flush exposed mucous membranes with water Use of antiseptics for wound care Injection of disinfectants into the wound Application of bleach to the wound	75 (65.8%) 97 (85.1%) 55 (48.2%) 21 (18.4%) 20 (17.5%)
When should post exposure prophylaxis be initiated after an exposure incident?	>72 hours Within 72 hours I don’t know	6 (5.3%0 104 (92.0%) 3 (2.7%)
What is the duration of post exposure prophylaxis?	2 weeks 4 weeks 3 months I don’t know	10 (8.8%) 48 (42.1%) 19 (16.7%) 37 (31.6%)

1 n (%).

a multiple answers allowed.

Regarding immediate first aid for exposure, practices such as washing skin injuries with soap and water (65.8%) were widely recognized, while fewer endorsed inappropriate measures like injecting disinfectants (18.4%) or applying bleach (17.5%) (Table 2). Timing in PEP is well-understood, with 92% of respondents aware that initiation should occur within 72 hours. However, 77.2% of respondents were unable to identify the regimen in use. Similarly, uncertainty surrounds the recommended duration for PEP, with only 42.1% of respondents aware that PEP should continue for four weeks (Table 2).

The knowledge score was calculated based on responses to nine key questions assessing awareness to HIV PEP, sources of information, understanding exposure incidents, first aid procedures, timing and duration of PEP, and knowledge of the recommended PEP drug regimens. Correct responses were awarded one point each, with a total possible score of 9 points. Participants who scored 7 or more points were categorized as having ‘good knowledge’, while those who scored below 7 points were categorized as having ‘poor knowledge’. Majority (75.4%) had good knowledge of HIV PEP (Table 2). Among the respondents, 62.3% report awareness of a PEM (postexposure management) protocol in their institution, while a notable 33.3% are uncertain, and 3.5% are unaware of any existing protocol.

Also, nearly half (49.1%) of the respondents reported experiencing a needlestick injury or similar exposure event, with the median frequency of incidents being one in the past year. Of those who had been exposed, just over half (53.6%) reported the incident to their institution (Table 3). The compliance level with HIV PEP among exposed respondents is generally low, with only 41.1% demonstrating good compliance and 58.9% exhibiting poor compliance (Table 3).

**Table 3.** Compliance with HIV PEP

Characteristic	N = 56^1^
**Times pricked in last one year**	1.00 (0.00, 1.00)
**Incident reported**	30 (53.6%)
**Asked patient about blood borne viruses**	44 (78.6%)
**Requested blood tests for the patient**	32 (57.1%)
**HIV status of source patient** Positive Negative I don’t know	1 (1.8%) 41 (73.2%) 14 (25.0%)
**Commenced post exposure prophylaxis for HIV**	2 (3.6%)
**Initiation time for post exposure prophylaxis** Within 24 hours after exposure incident >36 hours postexposure	1 (50.0%) 1 (50.0%)
**Compliance level** Good compliance Poor compliance	23 (41.1%) 33 (58.9%)

1 n (%); Median (IQR).

Compliance to post exposure management protocols was assessed based on five key actions: reporting the incident, asking the patient if they carried blood-borne viruses, requesting blood tests for the patient, initiating PEP for HIV, and ensuring that PEP was commenced within 24 hours of exposure. Each correct action was assigned one point, with a total possible score of 5 points. Participants who scored 3 or more were categorized as having ‘good compliance’, while those who scored below 3 were categorized as having ‘poor compliance’. Only 41.1% of respondents had good compliance [Table 3].

Finally, professional cadre does not significantly influence compliance, as residents, who make up the majority of both compliance categories, represent 30.4% of the good compliance group and 37.5% of the poor compliance group (*P* = 0.7) [Table 4]. House officers and consultants show similarly balanced representation across both compliance groups, indicating that level of clinical experience or professional responsibility does not necessarily correlate with better adherence to PEP protocols.

**Table 4.** Association between cadre of dental practitioner and compliance with HIV PEP

Characteristic	Good compliance, N = 23^1^	Poor compliance, N = 33^1^	*p*-value^2^
Cadre of Practitioner			0.7
Consultant	3 (5.4%)	4 (7.1%)	
House Officer	3 (5.4%)	8 (14.3%)	
Resident	17 (30.4%)	21 (37.5%)	

1 Median (IQR); n (%).

## Discussion

The current study found a high level of knowledge (75.4%) among respondents regarding factors associated with exposure incidents and postexposure management. These findings align with a Brazilian study, which reported that over 94% of dental surgeons were well-informed about occupational postexposure protocols.^15^ However, this contrasts with a study from Iran, which observed significantly lower knowledge levels among dentists concerning exposure to blood and body fluids.^16^ The disparity in knowledge levels across these studies may be attributed to differences in healthcare settings and regional factors, highlighting the potential influence of local contexts on occupational health awareness among dental professionals.

Of those who had experienced occupational exposures, approximately 53.6% reported the incidents, with a similar proportion requesting blood tests for the involved patients. This reporting rate differs from international findings, such as the significantly lower rates (5–25%) observed among Croatian dentists.^17^ In Cameroon, 62% of healthcare workers who experienced accidental blood exposure did not report it.^15^ Conversely, a study in eastern Ethiopia documented a higher reporting rate of 79.2% among healthcare workers.^18^ These disparities may be attributed to regional differences in healthcare systems, organizational cultures, and reporting protocols. The relatively higher reporting rate in this Nigerian study, compared to some international findings, suggests potential variations in awareness, institutional policies, or reporting attitudes in the local dental settings. However, it’s noteworthy that a substantial proportion of exposure incidents remained unreported, aligning with global trends in healthcare settings.

The present study revealed that while postexposure management (PEM) protocols were established in both investigated institutions, awareness and implementation among dental practitioners varied significantly. Over half of the respondents acknowledged the existence of PEM protocols, but a notable 33.3% were uncertain, and 3.5% were completely unaware. These findings align with a study in a Central African regional hospital, where 55% of healthcare workers were aware of existing PEM facilities, indicating a broader trend of moderate awareness across African healthcare settings.^15^ This consistency across different African healthcare settings indicates a broader trend of moderate awareness levels regarding PEM protocols. Importantly, only 29.1% of respondents in the current study reported routine adherence to PEM protocols, suggesting that even when protocols exist, they may not be effectively integrated into daily clinical practices or emphasized within the institutional culture.

This study revealed a concerning trend in HIV PEP compliance among dental practitioners, with only 41.1% demonstrating good adherence to PEP protocols, while 58.9% showed poor compliance. This finding underscores a critical area for improvement, as proper PEP adherence is crucial for reducing HIV transmission risk following occupational exposure. The observed compliance level is consistent with previous research in similar healthcare settings, such as a study in Benin City, Nigeria, where only 36% of exposed dental surgeons took PEP.^19^ This similarity suggests that suboptimal PEP compliance may be a widespread issue, particularly in developing countries. More research needs to be conducted into factors that may be associated with the low compliance rate despite a relatively good knowledge of PEP among these health professionals. A qualitative study may provide more insight into this and aid development of policy interventions to improve compliance rate.

## Conclusion

This study highlights that dental practitioners in Nigerian tertiary hospitals, while highly aware of HIV postexposure management, exhibit significant gaps in their knowledge of specific protocols and compliance with postexposure procedures. Future research should explore factors contributing to the discrepancy between awareness and practice. Comparative studies across various healthcare settings in different regions of Nigeria and other developing countries, focusing on dental practitioners, dental assistants, and dental students, could provide valuable insights. These investigations should aim to identify best practices for clinicians in implementing postexposure protocols, hospital educators in designing effective training programs, and infection control departments in establishing and monitoring adherence to postexposure management guidelines. Such research would contribute to enhancing HIV prevention strategies in dental healthcare settings across developing nations.
